# Surgical Management of Glaucoma Secondary to Bilateral Acute Iris Transillumination: A Role for Gonioscopy-assisted Transluminal Trabeculotomy

**DOI:** 10.18502/jovr.v16i1.8258

**Published:** 2021-01-20

**Authors:** Stephanie Wey, Jason Flamendorf, Sapna Sinha, Daniel Lee

**Affiliations:** ^1^Department of Ophthalmology, University of Cincinnati, OH, USA; ^2^Glaucoma Service, Wills Eye Hospital, PA, USA

**Keywords:** Bilateral Acute Iris Transillumination, Fluoroquinolone, Glaucoma, Ocular Hypertension, Gonioscopy-assisted Transluminal Trabeculotomy

## Abstract

**Purpose:**

We report a case of bilateral acute iris transillumination (BAIT) in a young woman associated with ocular hypertension which eventually progressed to glaucoma that was treated with gonioscopy-assisted transluminal trabeculectomy (GATT).

**Case Report:**

A 37-year-old otherwise healthy female presented with intermittently red and inflamed eyes and blurred vision. She was treated with oral moxifloxacin months prior to presentation. Iris transillumination defects, a pigmented anterior chamber reaction, the absence of keratic precipitates, and a history of upper respiratory infection treated with an oral fluoroquinolone prompted the diagnosis of BAIT. Intraocular pressure (IOP) remained uncontrolled on multiple glaucoma medications. Following the development of new visual field defects, indicating progression to glaucoma, GATT with cataract extraction was performed.

**Conclusion:**

Although surgical intervention is rare with BAIT, our case demonstrates that GATT may be used effectively in those patients needing better IOP control before considering incisional glaucoma surgery.

##  INTRODUCTION 

Bilateral acute depigmentation of the iris (BADI) and bilateral acute iris transillumination (BAIT) are recently described clinical diagnoses of uncertain etiology that tend to occur in young women and may be associated with viral illness and/or fluoroquinolone use.^[1,2]^ BADI consists of a predominantly pigmented anterior chamber (AC) reaction and bilateral, usually symmetric, depigmentation of the iris stroma without transillumination defects (TIDs). It tends to be self-limited with resolution of iris depigmentation and no effect on intraocular pressure (IOP).^[1,3,4]^ BAIT also produces pigment in the AC but is characterized by TIDs, variable pupillary sphincter paralysis, and an increased likelihood for IOP elevation.^[2]^ Seventy-nine cases of BAIT have been reported in the literature since the first description of the syndrome in 2004.^[5]^ We present a case of BAIT with secondary open-angle glaucoma and uncontrolled IOP that was ultimately managed with gonioscopy-assisted transluminal trabeculotomy (GATT), which to our knowledge is unprecedented.

##  CASE REPORT

**Figure 1:**
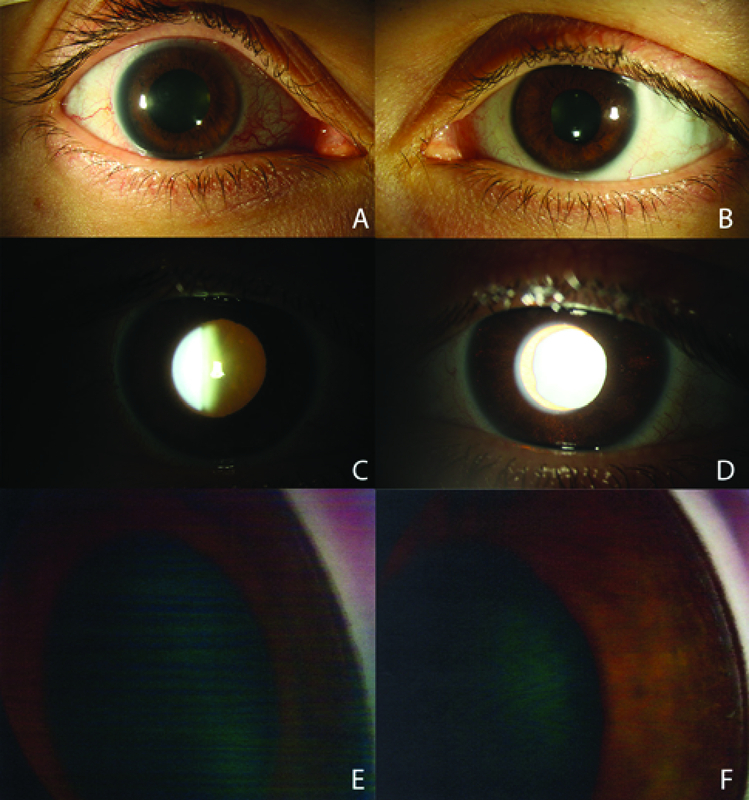
(A & B) External photos of the right and left eyes. The right eye has a mydriatic pupil with pupillary sphincter paralysis and nasal conjunctival injection. (C & D) Retroillumination photos of the right and left eyes. The right eye has a reduced red reflex, likely due to a more significant cataract. Visible in the left eye are diffuse, moth-eaten transillumination defects. (E & F) Gonioscopy photos of the right and left eyes showing open angles with dense trabecular meshwork pigmentation.

A 37-year-old otherwise healthy female was referred to our glaucoma clinic for uncontrolled IOPs in the setting of bilateral hypertensive uveitis. Her initial symptoms included intermittent red and inflamed eyes accompanied by blurred vision. She was managed by several ophthalmologists for five months prior to presenting at our clinic. She had been diagnosed with bilateral hypertensive uveitis, for which she was placed on and off topical steroids and glaucoma drops. Her symptoms notably began shortly after taking a 10-day course of oral moxifloxacin (Avelox) for sinusitis. Initial and maximum recorded IOPs were 30 mmHg in the right eye and 17 mmHg in the left eye. At presentation to our clinic, visual acuity was 20/60 and 20/25 with –2.25 sphere and –1.50 sphere in the right and left eyes, respectively. Both pupils were poorly reactive to light, and the left pupil was noted to have an oval shape [Figure 1A–D]. IOPs by Goldmann Applanation Tonometry were 19 mmHg and 9 mmHg in the right and left eyes, respectively, on topical latanoprost and fixed-combination dorzolamide-timolol in both eyes, brimonidine in the right eye, and oral methazolamide. Lids and lashes were unremarkable. Cornea exam revealed a bilateral, diffuse endothelial pigmentation. The AC was deep in both eyes and showed trace flare in the right eye and rare pigmented cell without flare in the left eye. Gonioscopic exam was open scleral spur with a flat iris contour and heavy pigment deposition in both eyes [Figure 1E–F]. The irides demonstrated diffuse, patchy TIDs in both eyes. There was trace nuclear sclerosis with a 1+ posterior subcapsular (PSC) cataract in the right eye, and a trace nuclear sclerosis cataract in the left eye. On dilated fundus examination, the right and left optic nerves had cup-to-disc ratios of 0.4 and 0.1, respectively, with sloping of the superior rim of the right optic nerve. The remaining fundus exam was unremarkable.

**Figure 2:**
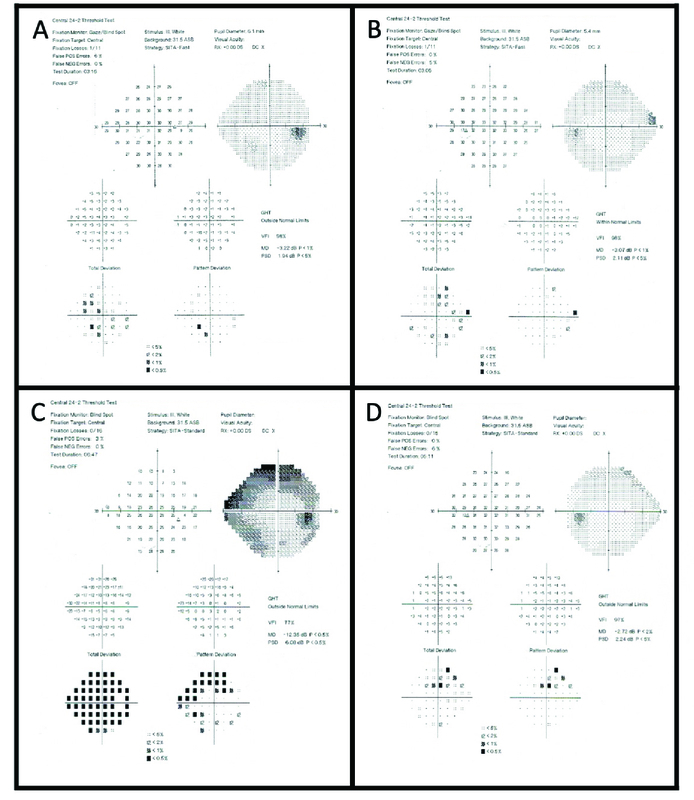
(A & B) 24-2 Humphrey visual field of right and left eyes at initial presentation. (C & D) 24-2 Humphrey visual field of right and left eyes five months after initial presentation to our clinic showing superior arcuate and inferior nasal defects in the right eye.

**Figure 3:**
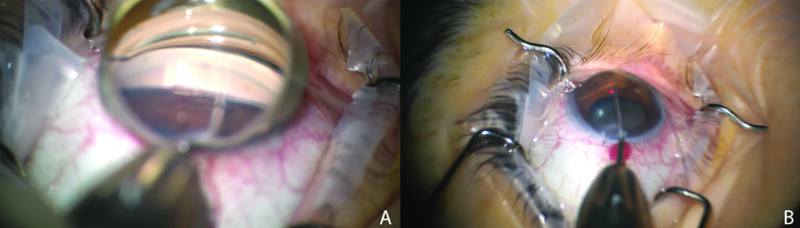
(A) Intraoperative gonioprism view demonstrating insertion of microcatheter into Schlemm's canal using microsurgical forceps via a goniotomy at the nasal angle. (B) The microcatheter has a red light at its proximal end, which is visible through the sclera as the microcatheter passes through Schlemm's canal. Once it has been passed for 360 degrees, the proximal end is held in place while the distal end is externalized to create a 360-degree trabeculotomy.

Optical coherence tomography of the retinal nerve fiber layer (OCT-RNFL) demonstrated mild thinning in the right eye with a corresponding superior nasal step on 24-2 Humphrey visual field (HVF) [Figure 2A]. The OCT-RNFL and HVF in the left eye were within normal limits [Figure 2B]. A prior work-up with a uveitis specialist yielded negative results for syphilis, Lyme, and HLA-B27 antigen.

The patient's acute presentation associated with heavy AC pigmentation, diffuse iris TIDs, prior upper respiratory infection (URI), and oral fluoroquinolone use led to the diagnosis of BAIT.

IOPs were initially maintained on her presenting medical therapy. During follow-up visits, the patient experienced intermittent redness and photophobia, which were treated with topical steroids. The AC reaction remained predominantly pigmented with an absence of keratic precipitates. IOPs were labile but remained well-controlled on and off oral acetazolamide. Five months following her initial diagnosis, the IOP of the right eye was 28 mmHg despite maximum medical therapy. A repeat HVF revealed significant progression of the field defects in the right eye [Figure 2C]. The pressures remained controlled with full fields in the left eye [Figure 2D]. The PSC cataract had worsened, and her best-corrected visual acuity had declined to 20/100.

Due to the visually significant cataract and elevated IOP uncontrolled with medications, a combined cataract extraction and gonioscopy-assisted transluminal trabeculotomy (GATT) was performed in her right eye using a fiberoptic microcatheter (iTRACK, Ellex, iScience Inc., Fremont, CA) as previously described by Grover and colleagues [Figure 3].^[6]^ IOP on the first postoperative day improved to 12 mmHg on dorzolamide-timolol and pilocarpine. Following resolution of inflammation with tapering doses of topical steroids, pilocarpine was discontinued at the one-month follow-up. IOP remained controlled in the mid-teens. At her final follow-up eight months after the surgery, IOP was 9 mmHg in the right eye on dorzolamide-timolol alone and 10 mmHg in the left eye.

##  DISCUSSION

The differential diagnosis for anterior segment pigmentation and ocular hypertension is limited. Common diagnoses include pigment dispersion syndrome, herpetic uveitis, pseudoexfoliation, Uveitis-Glaucoma-Hyphema syndrome, and trauma. Less common diagnoses include iris/ciliary body melanomas, irradiation-induced depigmentation, and finally BADI/BAIT. Pigment dispersion syndrome was certainly considered, but the patient lacked the classic findings of posterior iris bowing and mid-peripheral TIDs. Furthermore, Krukenberg spindles, which signify a more indolent and chronic depigmentation process, were notably absent. This, in addition to the acuity of onset, severe and diffuse iris pigment loss, inflammatory symptoms, and preceding URI with oral fluoroquinolone use led to the unusual diagnosis of BAIT.

BAIT was first described in a series of five patients who presented with bilateral photophobia and injection 10 to 14 days after taking oral moxifloxacin.^[7]^ In the largest case series to date, all 26 patients examined presented with photophobia. Most had pigmented cells in the AC, bilateral diffuse iris TIDs, and mydriasis with poor pupillary sphincter function. Nearly three-quarters of the patients had a preceding URI, and nearly half of this subset had taken oral moxifloxacin.^[2]^ Our patient has many clinical features consistent with BAIT. The more patchy and milder TIDs than those described in other reported cases may be partly due to media opacity from the cataract in her right eye.

BADI is another rare, female-predominant clinical entity potentially linked to a prior URI and/or moxifloxacin use. Like BAIT, BADI typically presents with photophobia, bilateral involvement, and a pigmented AC reaction. The iris findings, however, are drastically different. Unlike BAIT, there is depigmentation of the iris stroma, yielding a greyish, granular appearance, a lack of TIDs, and a normal pupil.^[1]^ Another distinguishing feature between BAIT and BADI is the incidence of elevated IOP. Tugal-Tutkun and colleagues showed that 54% of patients with BAIT developed elevated IOP during their disease course, with 27% requiring oral acetazolamide and 8% requiring bilateral trabeculectomies with mitomycin C.^[2]^ In contrast, only one patient (4%) in the BADI cohort demonstrated elevated IOP. Gonioscopic findings were similar for both diseases, demonstrating heavy angle pigmentation, especially inferiorly.

Ocular hypertension is a common complication of BAIT.^[2,8,9,10]^ Patients with BAIT often receive topical steroids, which may contribute to the rise in IOP. However, ocular hypertension with BADI is rare despite steroid use, and elevated IOP in the setting of BAIT has been reported in the absence of steroids, suggesting a mechanism for ocular hypertension intrinsic to the disease.^[1,2][8]^ Surgical management in this condition is rare; early cases involved bilateral trabeculectomies.^[2]^ More recently, Trabectome was the first micro-incisional canal-based procedure to be utilized in the setting of BAIT.^[8]^ Unlike Trabectome, which is limited to the nasal angle, GATT allows for a circumferential treatment. This provides a theoretical advantage by exposing more collector channels and improving the likelihood for treatment success. GATT has been utilized in patients with secondary open-angle glaucomas and has shown a high success rate and robust IOP reduction through 24 months of follow-up.^[6]^ Our patient has demonstrated excellent IOP control after GATT.

In summary, to the best of our knowledge, this is the first reported case of GATT used to treat glaucoma secondary to BAIT. Although surgical intervention is rare with BAIT, our case demonstrates that GATT may be used effectively in those patients needing better IOP control before considering incisional glaucoma surgery.

## References

[1] Tugal-Tutkun I, Araz B, Taskapili M, Akova YA, Yalniz-Akkaya Z, Berker N, et al. Bilateral acute depigmentation of the iris: report of 26 new cases and four-year follow-up of two patients. *Ophthalmology* 2009;116:1552–1557. 10.1016/j.ophtha.2009.02.019 19545903

[2] Tugal-Tutkun I, Onal S, Garip A, Taskapili M, Kazokoglu H, Kadayifcilar S, et al. Bilateral acute iris transillumination. *Arch Ophthalmol* 2011;129:1312–1319. 10.1001/archophthalmol.2011.310 21987674

[3] Tugal-Tutkun I, Urgancioglu M. Bilateral acute depigmentation of the iris. *Graefe's Arch Clin Exp Ophthalmol* 2006;244:742–746. 10.1007/s00417-005-0137-x 16205935

[4] Atilgan CU, Kosekahya P, Caglayan M, Berker N. Bilateral acute depigmentation of iris: 3-year follow-up of a case. *Ther Adv Ophthalmol* 2018;10:2515841418787988. 10.1177/2515841418787988 PMC6056785 30046770

[5] Perone JM, Chaussard D, Hayek G. Bilateral acute iris transilumination (BAIT) syndrome: literature review. *Clin Ophthalmol *2019;13:935–943. 10.2147/OPTH.S167449 PMC6556534 31239635

[6] Grover, DS, Smith O, Fellman RL, Godfrey DG, Gupta A, Montes de Oca I, et al. Gonioscopy-assisted transluminal trabeculotomy: an ab interno circumferential trabeculotomy: 24 months follow-up. *J Glaucoma* 2018;27:393–401. 6 10.1097/IJG.0000000000000956 29613978

[7] Wefers Bettink-Remeijer M, Brouwers K, van Langenhove, L, De Waard PWT, Missotten TO, Martinez Ciriano JP, et al. Uveitis-like syndrome and iris transillumination after the use of oral moxifloxacin. *Eye * 2009;23:2260–2262. 10.1038/eye.2009.234 19851342

[8] Den Beste KA, Okeke C. Trabeculotomy ab interno with Trabectome as surgical management for systemic fluoroquinolone-induced pigmentary glaucoma: a case report. *Medicine *2017;96:e7936. 10.1097/MD.0000000000007936 PMC5671812 29068979

[9] Morshedi RG, Bettis DI, Moshirfar M, Vitale AT. Bilateral acute iris transillumination following systemic moxifloxacin for respiratory illness: report of two cases and review of the literature. *Ocul Immunol Inflamm* 2012;20:266–272. 10.3109/09273948.2012.670359 22694259

[10] Willermain F, Deflorenne C, Bouffioux C, Janssens X, Koch P, Caspers L. Uveitis-like syndrome and iris transillumination after the use of oral moxifloxacin. *Eye *2010;24:1419–1420. 10.1038/eye.2010.19 20379213

